# Patient-tailored azacitidine, venetoclax and gilteritinib triplet therapy appear to improve tolerability while preserving encouraging clinical outcomes in *FLT3*-mutated relapsed/refractory AML

**DOI:** 10.1007/s00277-026-07297-2

**Published:** 2026-10-23

**Authors:** Matilda Arvidsson Kvissberg, Christer Nilsson, Niklas Landberg, Albin Österroos, Aristea Papageorgiou, Bertil Uggla, Vladimir Lazarevic, Stefan Deneberg, Martin Jädersten

**Affiliations:** 1 Center for Hematology and Regenerative Medicine, Karolinska Institutet, Stockholm, Sweden; 2 Department of Hematology, Karolinska University Hospital, Stockholm, Sweden; 3 Division of Clinical Genetics, Department of Laboratory Medicine, Lund University, Lund, Sweden; 4 Department of Hematology, Oncology and Radiation Physics, Skåne University Hospital, Lund, Sweden; 5 Department of Medical Sciences, Uppsala University Hospital, Uppsala, Sweden; 6 Department of Medicine, Halland Hospital, Halmstad, Sweden; 7 Department of Medicine, Division of Hematology, Örebro University Hospital, Örebro, Sweden

**Keywords:** Targeted therapy, AML, Gilteritinib, Azacitidine, Venetoclax

## Abstract

*FLT3-*mutated acute myeloid leukemia (AML) is associated with high relapse rates and treatment resistance. Triplet therapy with azacitidine (AZA), venetoclax (VEN), and the FLT3 inhibitor gilteritinib (GILT) yields high response rates but is limited by myelosuppression and infections. We conducted a multicenter retrospective study of 29 *FLT3*-mutated AML patients treated with GILT-triplets. The primary endpoint was overall survival (OS); secondary endpoints event-free survival (EFS) and time to neutrophil and platelet recovery. Patients received one of three regimens: concomitant triplet therapy (Group 1); concomitant AZA + VEN followed by concomitant VEN+GILT (Group 2); or overlapping AZA + VEN followed by GILT monotherapy (Group 3). For the entire cohort median OS was not reached after more than five years of follow-up. High response rates were observed across all schedules. Limiting concomitant exposure reduced myelosuppression and decreased infectious complications compared with full triplet therapy. Median OS was not reached in Groups 1 and 2 after three years, whereas Group 3 had a median OS of 18 months. Median EFS was 40.5 months in Group 1 and was not reached at five years in Group 2, compared with 11.5 months in Group 3. Our data suggest that reducing the duration of triple-drug overlap may improve tolerability without compromising clinical outcomes.

**Supplementary Information:**

The online version contains supplementary material available at https://doi.org/10.1007/s00277-026-07297-2.

## Introduction

Fms-like tyrosine kinase 3 *(FLT3)* mutations occur in around 30% of patients with acute myeloid leukemia (AML), the most common being internal tandem duplication (*FLT3-ITD*) [1]. *FLT3* mutations are associated with highly proliferative disease, higher relapse rates [2], and are recognized drivers of treatment resistance in relapsed and refractory (R/R) AML [3].

The ADMIRAL study demonstrated that monotherapy with gilteritinib (GILT) in R/R AML resulted in superior overall survival (OS) compared to salvage chemotherapy [4, 5]. Real-world data reported comparable outcome; however, in the absence of allogeneic hematopoietic stem cell transplantation (HSCT), most patients relapse within one year [5–8]. The combination of the BCL2 inhibitor venetoclax (VEN) with the hypomethylating agent azacitidine (AZA) has significantly improved survival for patients who are ineligible for intensive chemotherapy [9, 10]. Despite this advance, relapse remains a concern, with *FLT3-ITD* mutation identified as a key driver of treatment resistance [3, 11].

To further enhance efficacy, triplet therapy with AZA, VEN and GILT has been explored, demonstrating high response rates and one-year OS of 26% in R/R AML and 83% in newly diagnosed (ND) AML [12]. However, this benefit has been offset by substantial hematological toxicity and infectious complications, [12, 13] with high mortality rate during remission [12]. Despite the high response rates, relapse still remains a challenge, particularly in R/R AML, where the median relapse free survival (RFS) was only 4.3 months [12]. This may be attributable to the limited tolerability, with the median number of cycles was only two in the R/R cohort [12].

We have explored three variants of GILT-based triplet regimens depending on the treatment objective and patient fitness. Here, we present real-world multi-center data on GILT-based triplet therapy in Sweden and propose that an individualized approach allows patients to remain on therapy longer, thereby potentially improving their outcome.

## Methods

### Patient population

We retrospectively analyzed AML patients (17 years old or older) with *FLT3* mutations from five Swedish centers, who had been treated with AZA, VEN, and GILT therapy. Patients with prior exposure to AZA, VEN and FLT3 inhibitors (including GILT) were included. The reasons for switching to triplet therapy were relapsed disease, de-escalation in patients unfit for further intensive chemotherapy, poor response to the first line therapy (refractory disease or only partial response), or progression from MDS to AML.

### Triplet regimens

**Fig. 1:**
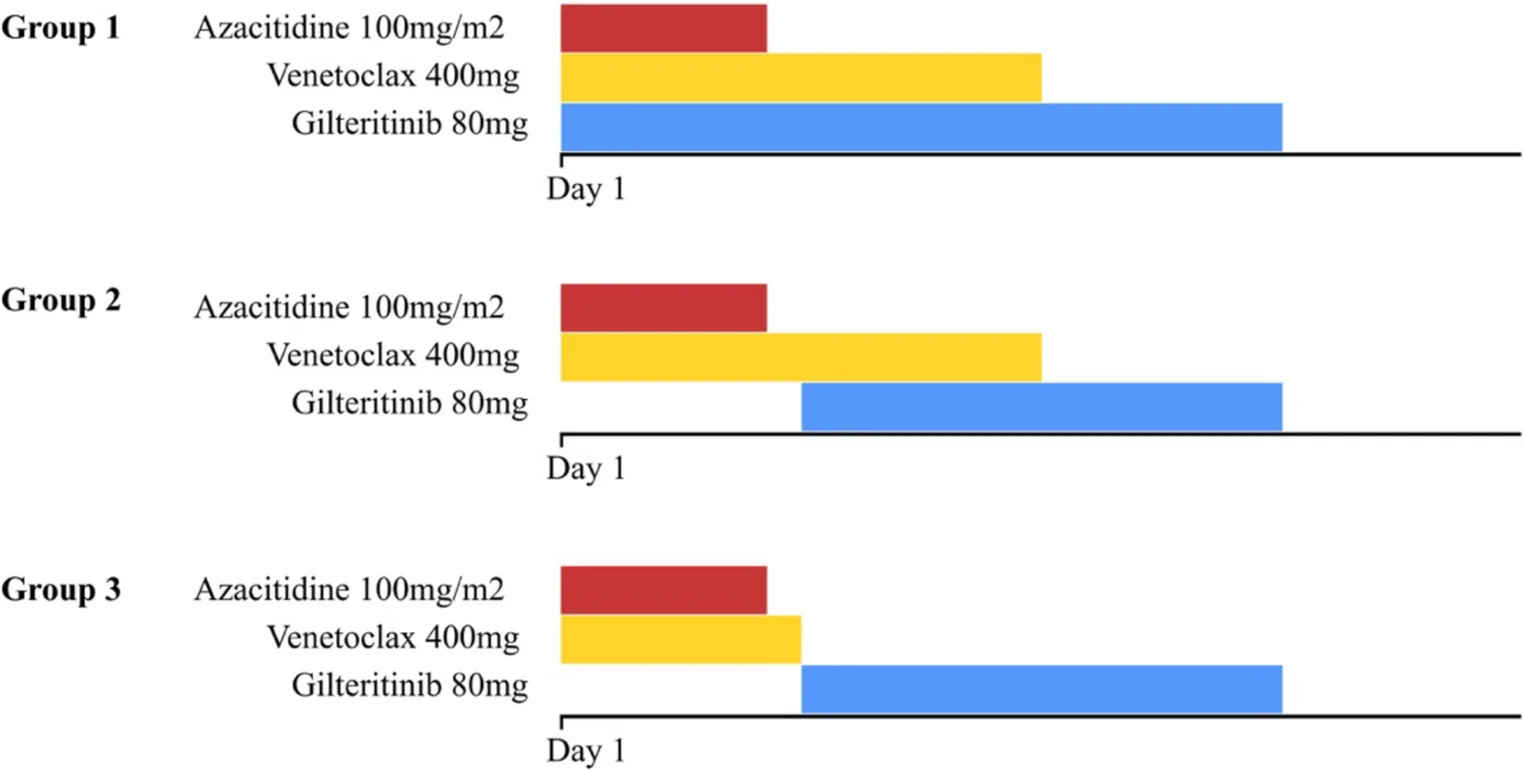
Schematic overview of the three GILT triplet regimes evaluated. Group 1: all three drugs were administered concomitantly from day 1 with AZA day 1 to day 5, VEN day 1 to day 7–21, GILT day 1 to day 8–28. Group 2: AZA and VEN were administered concomitantly during the first week, with subsequent overlap of VEN and GILT from day 6 or 8 to day 14–21. Group 3: AZA and VEN were administered concomitantly the first week, followed by GILT monotherapy from day 8 to day 15–28. Abbreviations: AZA, azacitidine; VEN, venetoclax; GILT, gilteritinib

AZA was administered at either 100 mg/m^2^ for five days (according to Swedish National Guidelines) or 75 mg/m^2^ for seven days. During cycle one, the dose of VEN was 400 mg daily with daily ramp-up of 100-200-400 mg, with dose reduction if concomitant administration of CYP3A inhibitors (e.g. posaconazole, fluconazole). The GILT dose was 80 mg daily. The duration of VEN and GILT exposure, as well as, the sequencing of AZA-VEN-GILT varied across patients and could broadly be categorized into three groups, Fig. 1. The initial patients were treated according to the Group 1 schedule. After observing substantial myelotoxicity, two modified schedules were developed (Group 2 and 3) at Karolinska University Hospital to improve tolerability. These regimens were used primarily in less fit patients, including non-transplant candidates or patients ineligible for further intensive chemotherapy. There were no formal institutional criteria for treatment allocation, instead, regimen selection was made at the discretion of the treating physicians.

### Response evaluation and definitions

Patients were evaluated by bone marrow examinations with assessment of measurable residual disease (MRD) on day 25–28 of cycle 1; no day 14 marrows were performed. Six patients did not follow the standard evaluation. Two patients with isolated myeloid sarcoma were evaluated only by PET CT, after cycle 1. Due to frailty, two patients were evaluated by peripheral MRD and two by bone marrow examination after cycle 2 and cycle 4, respectively. Subsequent bone marrow evaluations or peripheral blood genetic MRD assessments were performed as clinically indicated.

Response assessment, including MRD evaluation, was performed according to the European Leukemia Net 2022 guidelines [14], Supplemental Table S1. In addition, we utilized the term modified composite complete remission (mCRc), defined as CR, CRi, or MLFS. EFS was defined as time to hematological relapse (bone marrow blast of > 5%, reappearance of peripheral blasts in at least two blood samples, or extramedullary disease), treatment switch (due to rising MRD or treatment-related toxicity), or death.

### Outcome measurements

The primary outcome was OS. Secondary outcomes were EFS, and time to recovery of neutrophils (≥ 0.5 × 10^9^/l) and platelets (≥ 50 × 10^9^/L) after cycle one. OS and EFS were also analyzed based on disease status at start of triplet therapy.

### Ethical considerations

This retrospective chart review study was approved by the Swedish Ethical Review Authority (approval: Dnr 2024-07172-01). The study was conducted in accordance with the Declaration of Helsinki. All data were processed and stored in compliance with the General Data Protection Regulation (GDPR) and the Swedish Patient Data Act and were anonymized prior to analysis.

### Statistical analysis

D’Agostino & Pearson test for normality was used, and groups were compared using an unpaired t-test or one-way ANOVA for parametric data and the Kruskal-Wallis test for non-parametric data. To compare nominal variables, Fisher’s exact test was used. Remission duration, EFS, and OS were calculated using the Kaplan–Meier estimate, and differences between groups were assessed with the log-rank test. Data analyses were performed using Graph Pad Prism 11 (GraphPad Software Inc, Boston, MA).

## Results

### Patient characteristics at start of triplet therapy

29 patients were included from five centers: Karolinska University Hospital (*n* = 21), Lund University Hospital (*n* = 3), Uppsala University Hospital (*n* = 3), Örebro University Hospital (*n* = 1), and Halland County Hospital (*n* = 1). Group 1 (*n* = 13) received all three agents concomitantly from day 1. In this group, substantial myelosuppression was observed, particularly among older patients, prompting treatment modification with reduced drug overlap. Consequently, subsequent frail and older patients at Karolinska University Hospital were treated according to two newly developed schedules (*Methods* in Fig. 1), where AZA + VEN were given during the first week followed by VEN+GILT from day 8 (Group 2; *n* = 9), or AZA + VEN during the first week followed by GILT monotherapy from day 8 (Group 3; *n* = 7).

Baseline patient characteristics at the start of triplet GILT are presented in Table 1. Ten patients switched to GILT triplet due to insufficient response either to intensive chemotherapy or AZA + VEN. Nine patients de-escalated from intensive chemotherapy, as they were unfit for further intensive chemotherapy, primarily due to infectious complications. Three patients initiated the triplet therapy due to progression from MDS to AML, with two who progressed on single AZA. Seven patients received the GILT triplet therapy at relapse.

**Table 1:** Patients’ characteristics split over the three treatment regimens

		All patients	Group 1	Group 2	Group 3
Number of patients		29, *n* (%)	13*, *n* (%)	9**, *n* (%)	7**, *n* (%)
Age, median (range)		68 (17–83)	49 (17–83)	75 (39–83)	76 (38–77)
Sex	Female	14 (43)	7 (54)	3 (33)	4 (57)
ECOG	0	10 (34)	3 (23)	3 (33)	4 (57)
	1	13 (45)	6 (46)	4 (44)	3 (43)
	2	2 (7)	1 (8)	1 (11)	0
	3	4 (14)	3 (23)	1 (11)	0
ELN 2022^	Favorable	2 (7)	2 (15)	0	0
	Intermediate	16 (55)	7 (54)	4 (44)	5 (71)
	Adverse	11 (38)	4 (31)	5 (56)	2 (29)
ELN 2024^	Favorable	4 (14)	1 (8)	2 (22)	1 (14)
	Intermediate	24 (83)	12 (92)	6 (67)	6 (86)
	Adverse	1 (3)	0	1 (11)	0
Prior HSCT		5 (17)	2 (15)	2 (22)	1 (14)
*FLT3* mut.	TKD	6 (21)	2 (15)	3 (33)	1 (14)
	ITD	22 (76)	11 (85)	6 (67)	5 (71)
	both	1 (3)	0	0	1 (14)
Prior lines	1	10 (34)	0	6 (67)	4 (57)
	2	12 (41)	9 (69)	1 (11)	2 (29)
	>2	7 (24)	4 (31)	2 (22)	1 (14)
Disease state^^	CR/MLFS	10 (34)	4 (31)	3 (33)	3 (43)
	PR/NR	9 (31)	5 (38)	3 (33)	1 (14)
	R/MDS trans	10 (34)	4 (31)	3 (33)	3 (43)
Prior FLT3 TKI		20 (69)	11 (85)	4 (44)	5 (71)
Bridged to HSCT		8 (28)	6 (46)	2 (22)	0

*In Group 1, five patients are from the Karolinska University hospital and 8 patients from other hospitals. **All patients in Group 2 and 3 are from the Karolinska University Hospital. ^cytogenic risk group. ^^at start of triplet therapy. *Abbreviations: ECOG* Eastern Cooperative Oncology Group; *ELN* European Leukemia Net; *HSCT* hematopoietic stem-cell transplantation; FLT3, Fms-like tyrosine kinase 3; *ITD* internal tandem duplication; *TKD* tyrosine kinase domain; *TKI* tyrosine kinase inhibitor

Patients in Group 1 were younger, with a median age of 49 years, compared with 75 and 76 years in Group 2 and 3, respectively. Overall, 23 of 29 (79.3%) patients had *FLT3-ITD* mutation. Adverse cytogenic aberrations were present in 11 patients (37.9%), full mutational status shown in Supplemental Fig. S1. Mutational profile was analyzed at the start of triplet therapy for patients with relapsed AML or secondary AML (*n* = 3, MDS that progressed to AML). While, in patients that switched to the GILT triplet, either due to de-escalation or insufficient response to prior lines, mutational status was analyzed at diagnosis.

All Group 1 patients had received ≥2 prior lines of therapy, whereas most patients in Group 2 and 3 had received only 1 prior line (66.7% and 57.1% respectively). Details of prior therapies, disease status, and MRD status at treatment initiation are provided in Supplemental Table S2. Patients were stratified into three biologically relevant categories: mCRc, PR/NR and R/sAML. Relapsed and secondary AML were grouped together because both represent very high-risk disease entities with comparable poor outcome.

Prior FLT3 tyrosine kinase inhibitor (TKI) exposure was similar in Group 1 (84.6%) and Group 3 (71.3%) but lower in Group 2 (44.4%), and type of TKI inhibitor is provided in Supplemental Table S2.

### Triplet GILT as bridging to HSCT

Patients eligible for HSCT received triplet GILT as bridging to transplantation. In Group 1, nearly half of the patients were bridged to HSCT (*n* = 6, 46.2%; including one re-transplantation) and one patient received subsequent donor lymphocyte infusion (DLI), and one is scheduled for transplantation. In contrast, in Group 2 only two patients (12.5%) proceeded to HSCT and two received DLI, and no patients in Group 3. The patients that did not continue to HSCT were ineligible due to age and/or co-morbidities (*n* = 4).

Patients in Group 1 were generally fitter and eligible for a more myelosuppressive treatment and HSCT. In contrast, the regimens used in Group 2 and 3 were selected for frailer patients, considered unfit for concomitant GILT triplet. The two patients in Group 2 that did proceed to HSCT were deemed unfit for further intensive chemotherapy and concomitant triplet GILT at the time of treatment initiation but later recovered and were able to be bridged to transplantation. Characteristics of the eight transplanted patients are summarized in Supplemental Table S3.

### High remission rates and comparable survival across three different GILT triplet regimens

Treatment response was evaluated in 19 patients who were not in mCRc at time of treatment initiation, Table 2. In Group 2 and 3, true CR was achieved in 5 patients (50%) and the overall response rate of 100%. In Group 1 (*n* = 9), the overall response rate was likewise 100%, but only 2 patients were in true CR (22.2%). Among patients with available MRD assessments (14 of 19 patients; 73.7%), fewer than half were MRD negative in Group 1 (*n* = 3, 42.9%), compared with the majority in Group 2 and 3 (*n* = 6, 85.7%). Of the six patients in Group 1 who proceeded to HSCT, five patients underwent HSCT in a cytopenic state, with three still MRD positive. Serial MRD assessments are shown in Supplemental Fig. S2 and were done by RT-qPCR for *NPM1* (*n* = 12; were of 5 also were monitored for *FLT3* MRD in parallel), ddPCR for only *FLT3* (*n* = 3), by NGS (*n* = 2), by flow cytometry (*n* = 5). MRD was not assessed in 6 of the patients either due to high age (*n* = 3), fast progression of disease (*n* = 1) or not taken prior to HSCT (*n* = 2).

**Table 2:** Response to the GILT triplet therapy in patients not in mCRc at treatment initiation

Hematological response	All patients *n* = 19	Group 1 *n* = 9	Group 2 *n* = 6	Group 3 *n* = 4	*R*/sAML *n* = 10	PR/NR *n* = 9
mCRc	18 (94.7%)	9 (100%)	6 (100%)	3 (75%)	9 (90%)	9 (100%)
CR	7 (36.8%)	2 (22.2%)	3 (50.0%)	2 (50%)	2 (20%)	5 (55.6%)
CRi	8 (42.1%)	5 (55.6%)	2 (33.3%)	1 (25%)	6 (60%)	2 (22.2%)
MLFS	3 (15.8%)	2 (22.2%)	1 (16.7%)	0	1 (10%)	2 (22.2%)
PR	1 (5.3%)	0	0	1 (25%)	1 (10%)	0
NR	0	0	0	0	0	0
MRD						
Positive	5 (26.3%)	4 (44.4%)	0	1 (25%)	2 (20%)	2 (22.2%)
Negative	9 (47.4%)	3 (33.3%)	4 (66.7%)	2 (50%)	4 (40%)	5 (55.6%)
Not measured	5 (26.3%)	2 (22.2%)	2 (33.3%)	1 (25%)	4 (40%)	2 (22.2%)

Abbreviations: *mCRc* modified composite complete response, *CR* complete remission, *CRi* complete remission with incomplete count recovery, *MLFS* morphologic leukemia-free state, *PR* partial response, *NR* no response, *MRD* measurable residual disease, groups defined under Methods and in Fig. 1

**Fig. 2:**
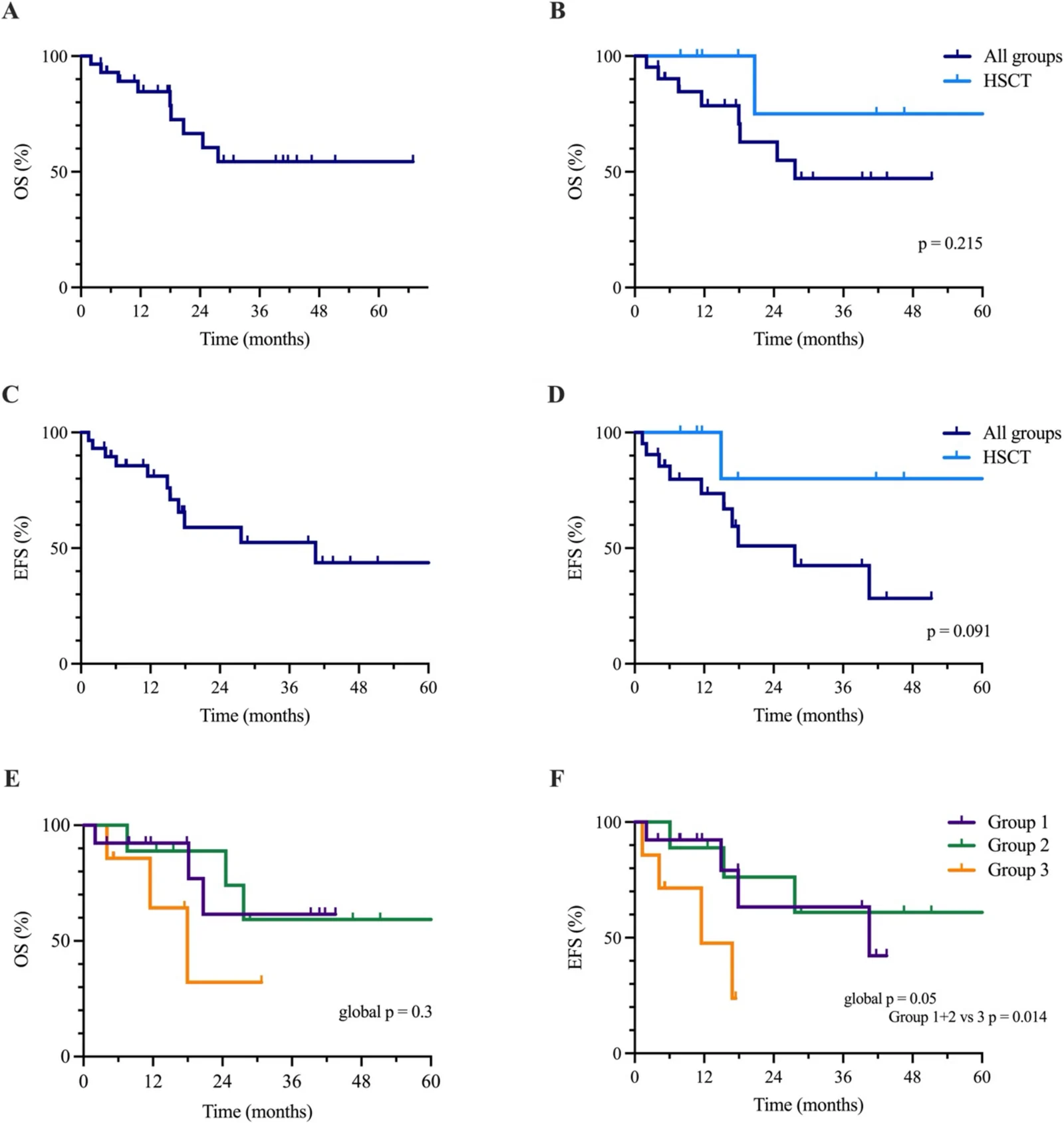
Survival outcomes. OS of all 29 patients treated with GILT triplet therapy (**A**). OS stratified by subsequent HSCT, compared using logrank analysis (**B**). EFS of all 29 patients treated with GILT triplet therapy (**C**). EFS stratified by subsequent HSCT, compared using logrank analysis (**D**). Outcome after GILT triplet therapy: (**E**) OS in the three treatment groups (as described in Fig. 1) compared using logrank analysis. (**F**) EFS in the three treatment groups, compared using logrank analysis. The groups were compared using logrank test. Abbreviations: OS, overall survival; EFS, event free survival; GILT, gilteritinib; HSCT, hematologic stem cell transplantation

For the entire cohort (*n* = 29), OS was 84.7% at 12 months and 78.6% at 18 months. Median OS had not been reached after five years (Fig. 2A), but in patients that were not transplanted the median OS was 27.6 months (Fig. 2B). The median EFS in the overall cohort was 40.5 months (Fig. 2C), with an estimated median beyond 5 years in transplanted patients and 27.6 months in non-transplanted patients (Fig. 2D). The median follow up was 30.8 months, with 30 days mortality at 0% and 60 days at 3.4%. In the 8 patients that relapsed half were *FLT3* positive at relapse, one patient had a *NPM1* MRD relapse with a subclonal *FLT3* at 0.005%, 3 were *FLT3* negative and 1 patient passed before marrow evaluation.

OS did not differ between the three treatment groups. Median OS was not reached in Groups 1 and 2 at the time of analysis, whereas Group 3 had a median OS of 18.0 months, potentially indicating an inferior outcome (Fig. 2E; *p* = 0.3). However, this is an exploratory analysis that cannot be used to draw firm conclusions. It is important to keep in mind the small sample size and the indication bias as patients in Group 3 were allocated this regime since clinician deemed them too frail for the Group 1 or 2 treatment regimes.

A statistically significant difference in EFS was observed among the groups (global *p* = 0.013). The median EFS was 40.5 months in Group 1 and not reached at five years in Group 2, while significantly shorter in Group 3 at 11.5 months (Group 3 vs. Group 1 + 2 *p* = 0.014 Fig. 2F). Again this should be viewed as an exploratory analysis and interpreted with caution, due to the previously mentioned limitations. In Supplemental Fig. S3 we show the OS and EFS in the non-transplanted patients per group compared to the patients that underwent HSCT. Our data indicates, as expected, that patients bridged to HSCT have superior outcome. However, more than half of non-transplanted patients in Groups 1and 2 had both median EFS and OS that exceeded two years.

### Inferior overall survival in patients with relapsed or secondary AML

We next evaluated treatment response and survival according to disease status at initiation of triplet therapy. Patients were categorized into the three groups: mCRc (CR/CRi/MLFS), PR/NR and R/sAML. Hematological response was assessed in the 19 patients that were not in mCRc at the start of the triplet therapy, Table 2. The overall response rate was 100%. Among patients with available MRD assessments, MRD negativity was observed in 9 of 13 patients (69.9%) with 5 patients achieving MRD negativity already after the first cycle, indicating a rapid and deep response.

**Fig. 3:**
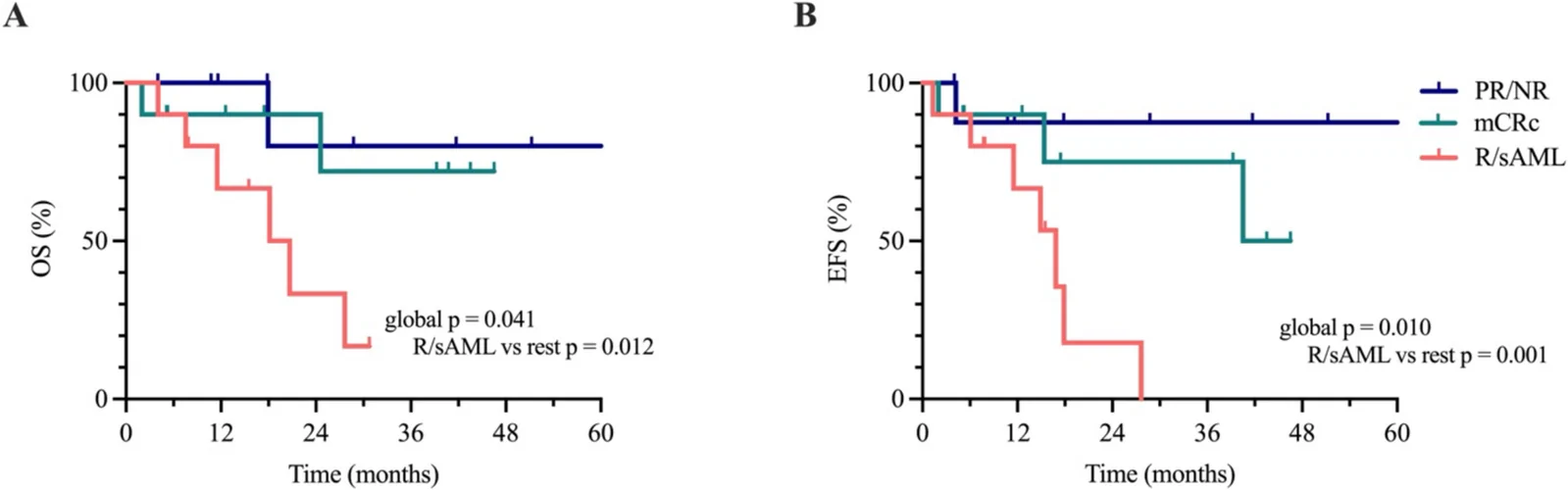
Comparison of outcome depending on the initial disease state. (**A**) Comparison of OS stratified by disease state at the start of GILT triplet therapy. (**B**) EFS according to disease state. The groups were compared using logrank test, global p value and the second p value comparing PR/NR and mCRc group to R/sAML. Abbreviations: OS, overall survival; EFS, event free survival; PR, partial response; NR, no response; mCRc, modified composite complete response; R, relapsed AML; sAML, secondary AML; R/R, relapsed/refractory

Despite similar response rates, OS and EFS differed markedly according to disease status. As expected, patients with R/sAML experienced inferior outcomes compared with the other groups, with a median OS of 19.4 months and a median EFS of 16.9 months (Fig. 3A-B, R/sAML vs. the rest *p* = 0.012 and *p* = 0.001 for OS and EFS, respectively). In contrasts, patients who were in PR/NR at the start of triplet therapy demonstrated excellent outcomes, with 80% OS and 87.5% EFS during the 5 years observational period. Favorable outcomes were also seen in patients who initiated triplet therapy in mCRc, with projected median OS extending beyond four years (Fig. 3A), and median EFS of 43.5 months (Fig. 3B).

### Treatment toxicity, dose adjustments, and cycle intervals

**Fig. 4:**
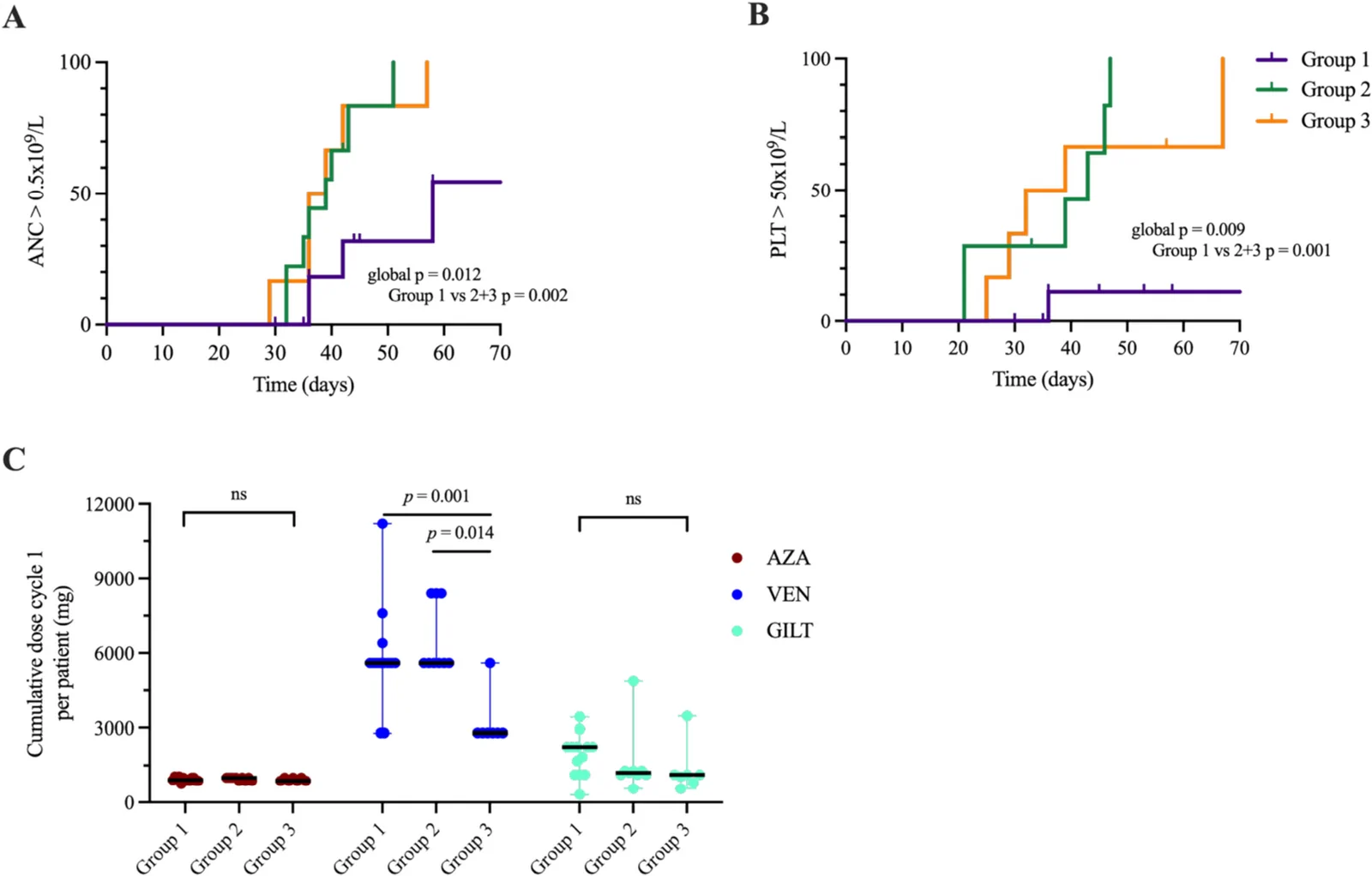
Count regeneration after GILT triplet therapy. Days until regeneration after cycle 1 of triplet therapy per group, ANC > 0.5 × 109/L (**A**) PLT > 50 × 109/L (**B**), data censored in patients that continued to HSCT or cycle 2 in a cytopenic state. The cumulative dose of the three drugs given in cycle one calculated as daily dose x number of days given (**C**). Abbreviations: ANC, absolute neutrophil count; PLT, platelet count, AZA, azacitidine; VEN, venetoclax; GILT, gilteritinib

When AZA, VEN, and GILT were administered concomitantly (Group 1), prolonged cytopenias were observed, resulting in infectious complications and delays in subsequent cycles, Supplemental Table S4. To assess the degree of myelosuppression, we compared the time to absolute neutrophil count (ANC) ≥ 0.5 × 10^9^/L and platelet count (PLT) ≥50 × 10^9^/L recovery. Time to recovery was censored at initiation of cycle 2 or HSCT, as some patients proceeded to subsequent therapy while still cytopenic. The estimated median time to ANC recovery after cycle one was 58 days in Group 1, compared to 39 days in Group 2 and 38 days in Group 3 (Fig. 4A, Group 1 vs. Group 2 + 3 *p* = 0.002). Most patients received granulocyte colony stimulating factor (G-CSF) during periods of neutropenia (cycle 1: *n* = 18, 62.1%). The estimated median time to PLT recovery was 105 days for Group 1, with only 11% of patients regenerating before day 60. In contrast, the median time to PLT recovery in Group 2 and 3 was 43 and 36 days, respectively (Fig. 4B, Group 1 vs. Group 2 + 3 *p* = 0.001). The cumulative dose of the three drugs in cycle 1 did not differ between the groups (Fig. 4C), apart from VEN dosage which was lower in Group 3. This suggests that it is the concomitant drug exposure that result in the extensive myelosuppression. When comparing the average cumulative drug exposure across all cycles, there was no difference between the Groups (Supplemental Fig. S4).

**Fig. 5:**
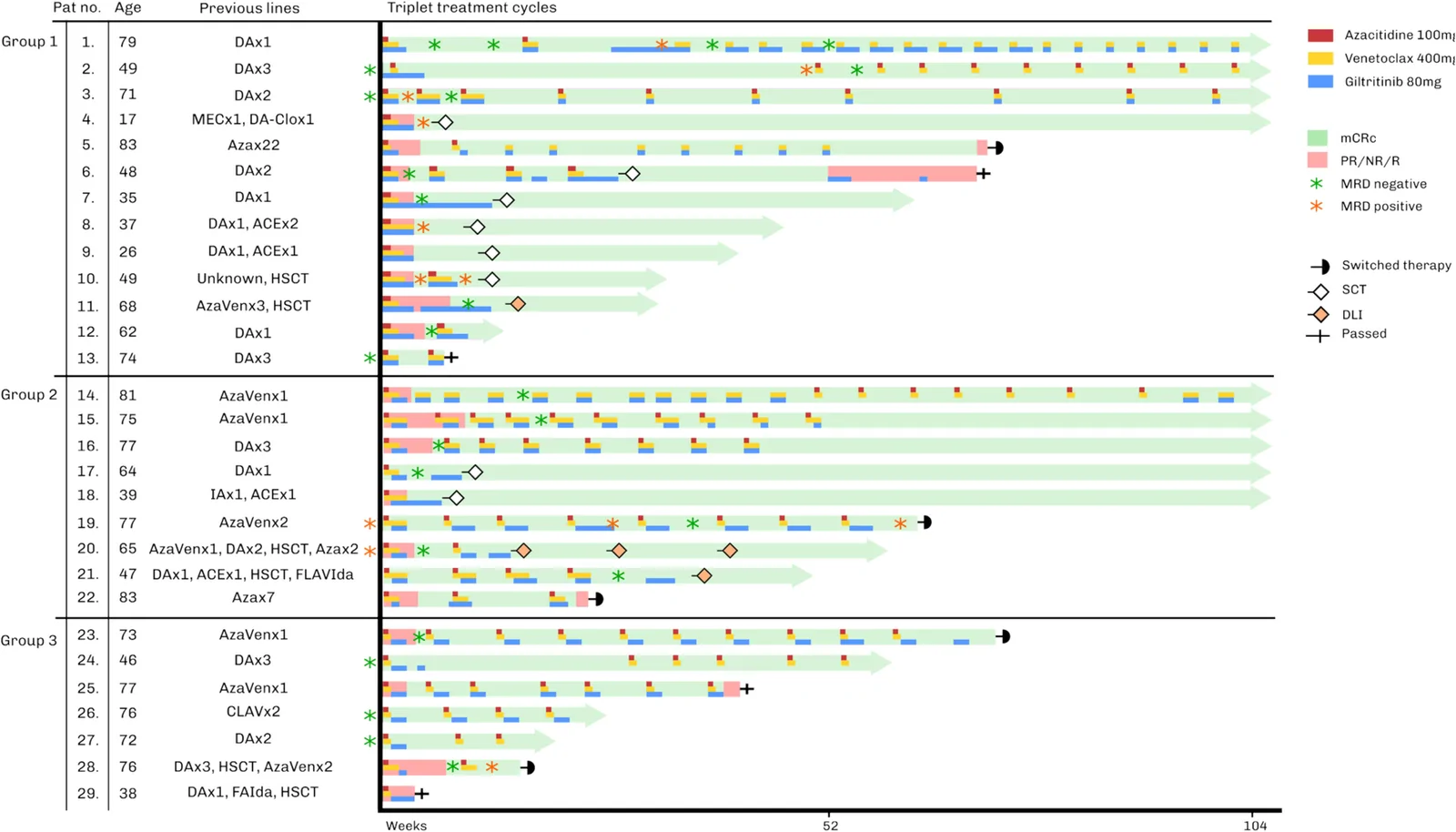
Swimmer plot overview of the three treatment groups, including administered cycles. Abbrevations: DA, daunorubicin and cytarabine; MEC, mitoxantrone, etoposide and cytarabine; Clo, clofarabine; Aza, azacitidine; ACE, amsakrine, cytarabine, etoposide; Ven, venetoclax; FLA-Ida, fludarabine, cytarabine, and idarubicin; FLAVIda, FLA-Ida + venetoclax; mCRc, modified composite complete response; PR, partial response; NR, no response; R, relapsed AML; MRD, measurable residual disease; HSCT, hematologic stem cells transplantation; DLI, donor lymphocyte infusion

When assessing cycle intervals, the time between cycles was longer in Group 1 compared to the other two groups (Fig. 5). Excluding the 11 patients who received triplet therapy as bridging to HSCT or DLI, only one patient in Group 1 was able to continue the concomitant triplet schedule beyond two cycles. To stay on the regimen this patient required dose reduction of VEN+GILT to 7 days per cycle and still needed 10–12 weeks between cycles (Patient 3, Fig. 5). The rest of the Group 1 patients de-escalated to doublet therapy (GILT + VEN or AZA + VEN) due to prolonged cytopenias (*n* = 3) or infectious complication (*n* = 1), except for one patient who died from neutropenic sepsis while in MRD negative CR after two cycles. Notably, one patient in Group 1 achieved MRD negativity after cycle one and de-escalated to GILT monotherapy due to extended cytopenias. During GILT monotherapy the patient became MRD positive but regained MRD negativity after escalating to a VEN+GILT doublet (Patient 1, Fig. 5). The median number of cycles for Group 1 was 2 (range 1–12, Supplemental Figure S5).

In contrast, several patients in Group 2 were able to remain on triplet therapy for extended periods, with dose adjustments implemented prior to subsequent cycles, to mitigate toxicity. The median number of cycles for Group 2 was 4 (range 1–10, Supplemental Figure S5). Two patients in Group 2 who achieved MRD-negative responses interrupted treatment after 8 and 10 cycles, respectively, because of cytopenias and subsequently underwent MRD monitoring. Of these patients, one relapsed after 18 months, while the other remains MRD negative after three years of treatment-free remission.

In Group 3, the median number of cycles was 1 (range 1–11; Supplemental Figure S5). Four patients in Group 3 only received one cycle of GILT triplet; one did not respond and passed away shortly after, two switched to AZA + VEN due to cytopenias and *FLT3* negativity, and the fourth one, in MRD negative remission, switched to MRD monitoring due to an infection and suspected GILT induced heart failure.

The most common grade ≥ 3 adverse event was febrile neutropenia. After cycle 1, febrile neutropenia occurred in 69.2% of patients in Group 1 (*n* = 9), 11.1% in Group 2 (*n* = 1), and none in Group 3. Additional adverse events included gastrointestinal bleeding (*n* = 2), atrial fibrillation (*n* = 2), heart failure (*n* = 1), and tumor lysis syndrome (*n* = 1). One patient discontinued GILT due to posterior reversible encephalopathy syndrome (PRES). A full summary of adverse events is provided in Supplemental Table S4.

## Discussion

In this multicenter retrospective study, we evaluated the feasibility, toxicity, and efficacy of AZA-VEN-GILT triplet therapy in *FLT3*-mutated R/R AML patients in Sweden. Consistent with previous reports [12, 13, 15], concomitant administration of all three agents (Group 1) had high efficacy but was associated with pronounced myelosuppression and a high incidence of infections. To make the treatment more tolerable in frail patients, we developed two alternative strategies in which no more than two components of the triplet regimen were given concomitantly (Groups 2 and 3, illustrated in Fig. 1). These modified treatment schedules appeared to have improved tolerability while maintaining encouraging clinical outcomes.

Patients in Group 1, with the most intensive triplet regimen, had a lower median age and were more heavily pretreated compared with Groups 2 and 3. In addition, the treatment goal for patients in Group 1 was more often bridging to HSCT. These substantial differences make it difficult to compare the groups. However, without a difference in cumulative dose of the three drugs in cycle 1, patients in Group 1 experienced a median time to ANC recovery of 58 days, while in contrast, with our novel mitigated strategies (Groups 2 and 3) the median time to ANC recovery was under 40 days. Only one patient in Group 1 was able to remain on the initial schedule beyond two cycles, while most patients in Group 2 and 3 could continue their initial regimens. Some elderly patients required de-escalation to doublet therapy to limit myelosuppression, while carefully being monitored for signs of disease progression.

No significant differences in OS were observed between the three triplet strategies, although EFS was higher in Group 1 and 2 compared with Group 3 (Fig. 3), suggesting potential combinatorial effects of both AZA + VEN and VEN+GILT; Group 3 had no overlap between VEN and GILT. However, it is important to keep in mind that patients in Group 3 were considered too frail for the other treatment regimens making direct comparisons challenging. Additionally, being a retrospective study comparing unbalanced sub-cohorts, we cannot confidently comment on the superiority or inferiority of any of the three regimes, a prospective randomized trial is needed to evaluate this further. An additional limitation in this real-world data study, is that heterogeneity of the response and MRD assessment, making it further difficult to compare the results.

Clinical outcomes of this study were encouraging in all three treatment groups, with a median OS in the entire cohort not reached after five years of follow-up. When censoring patients bridged to transplantation, the median OS was 27.6 months. Among the 19 patients who were not in remission at the initiation of triplet therapy, the median OS and EFS were 27.6 months (Supplemental Figure S6). These outcomes compare favorably with previously reported data in GILT-based triplet regimens, including the pivotal trial by Short et al. where the median OS and EFS in the R/R cohort was 5.8 and 4.3 months, respectively [12]. It is conceivable that this can be attributed to the improved tolerability of these regimes, allowing patients to stay longer on their triplet therapy.

In a subset of 9 patients who de-escalated from intensive chemotherapy to GILT triplet therapy, the 3-year OS was 70% and the single patient that continued to HSCT did not relapse after transplantation. This indicates that de-escalation from intensive chemotherapy to a target triplet regimen may be a viable novel approach in patients unfit to tolerate further heavy treatment, and it would be of interest to explore this strategy in a prospective setting.

Several efforts have been made to improve the tolerability of triplet therapies where a hypomethylating agent is combined with VEN and a FLT3 inhibitor. Recent recommendations from MD Anderson suggest a schedule with AZA 75 mg/m^2^ for 7 days, VEN 400 mg for 14 days and either GILT 80 mg for 14–28 days, with early discontinuation of VEN and the FLT3 inhibitor if the day 14 bone marrow assessment shows blast clearance [15]. This closely resembles our study subset Group 1, and we question the clinical utility of the day 14 bone marrow assessment given the near universal response rate.

We cannot give recommendations based on our small retrospective cohort. However, at Karolinska University Hospital our current routine in fit transplant candidates is to treat according to our Group 1 schedule: AZA 100 mg/m2 days 1–5 or 75 mg/m2 days 1–7 days, VEN 400 mg days 1–14 and GILT 80 mg days 1–21.

The pronounced hematological toxicity observed with the Group 1 triplet schedule indicates that concurrent administration of all three agents should be used with caution in frail patients. In non-transplant candidates, we currently use the Group 2 schedule: AZA 100 mg/m2 days 1–5 or 75 mg/m2 days 1–7, VEN 400 mg days 1–14 and GILT 80 mg day 8 to day 14 or 21. It remains unresolved from our data if the Group 3 regimen would be preferable in the most frail patients. In this setting, a dose-reduced version of the Group 2 schedule may also be viable alternative that preserves the synergistic effect of concurrent VEN-GILT.

In all groups, dosing during subsequent cycles were adjusted according to the tolerability and treatment goal to avoid excessive toxicity during remission. Given the marked patient heterogeneity and variability in myelotoxicity associated with the different GILT triplets, we consider flexible, patient-tailored dosing to be essential to enable prolonged treatment while maintaining an acceptable safety profile, particularly in frail older patients.

MRD monitoring is valuable to assess depth of response and to guide potential treatment de-escalation or escalation depending on the MRD trajectory. At treatment failure, reassessment of *FLT3* mutational status is recommended, as the relapse may be *FLT3*-negative in a significant number of patients [12].

## Conclusion and future directions

Consistent with previously reported data, our findings indicate that AZA-VEN-GILT triplet therapy has substantial anti-leukemic activity in *FLT3*-mutated AML, although significant myelosuppression frequently occurs, in particular when all three drugs are administered concomitantly. The retrospective design, small sample size, and heterogeneity in patient characteristics are clear limitations of this study, restricting the ability to draw conclusions regarding the comparative efficacy of the three triplet regimens. Nevertheless, these real-world data suggest that an individualized approach, in which the triplet regimens are adapted according to the patients and their treatment goal, may enhance tolerability without compromising clinical outcomes. Prospective, randomized studies are ongoing (MyeloMATCH - NCT06317649 and SEQUENCE trial - NCT06696183) to further define optimal dosing and scheduling. In the future, patient-tailored GILT-based triplet therapy may become an important addition to the therapeutic arsenal for patients with *FLT3*-mutated AML.

## Supplementary Information

Below is the link to the electronic supplementary material.

Supplementary Material 1

## Data Availability

The author confirms that all data generated or analysed during this study are included in this published article.
