# Supplementary material for: Patient-tailored azacitidine, venetoclax and gilteritinib triplet therapy appear to improve tolerability while preserving encouraging clinical outcomes in FLT3-mutated relapsed/refractory AML
Source: Ann Hematol. 2026 Oct 23;105(11):479. doi: 10.1007/s00277-026-07297-2 (PMC13694287; doi:10.1007/s00277-026-07297-2)
Supplement: Supplementary file 1 [file 277_2026_7297_MOESM1_ESM.pdf]

Supplemental data

Supplemental Table S1

Complete remission (CR):	Bone marrow blasts below 5%; absence of circulating blasts; absence of extramedullary disease; ANC ≥ 1.0x109/L; platelet count ≥ 100x109/L.
Complete remission with incomplete count recovery (CRi):	All CR criteria expect for residual neutropenia ANC < 1.0x109/L or platelet count < 100x109/L.
Morphologic leukemia-free state (MLFS):	Bone marrow blasts < 5%; absence of circulating blasts; absence of extramedullary disease; no hematologic recovery required.
Partial remission (PR):	All hematologic criteria of CR; decrease of bone marrow blast percentage to 5% to 25% and decrease of pre-treatment bone marrow blast percentage by at least 50%.
No response (NR):	Patients evaluable for response but not meeting the criteria for CR, CRh, CRi, MLFS or PR are categorized as having no response prior to the response landmark. Patients failing to achieve response by the designated landmark are designated as having refractory disease.

Supplemental Figure S1. Mutation profile according to patient groups. Molecular profiles were analyzed at initiation of triplet therapy for secondary AML and relapsed disease status, and at diagnosis for refractory patients, as well as, patients that de-escalated from intensive chemotherapy.

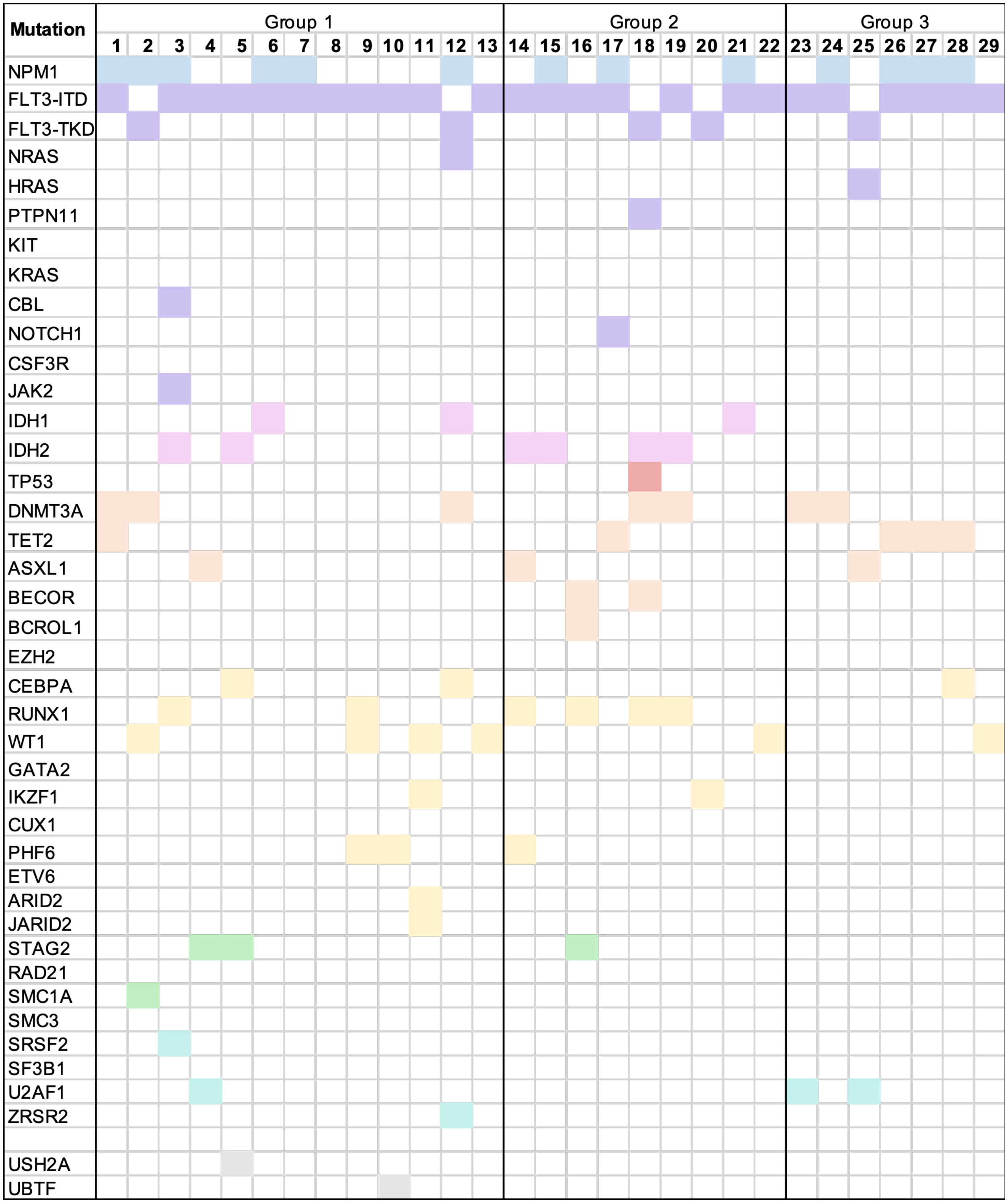

Supplemental Table S2

Group	Pat number	Age	ELN 2022 risk group	Previous treatment	Previous FLT3 TKI	At start of triplet GILT	
						BM status	MRD status
1	1	79	intermediate	DA x1	No	CR	Not measured
	2	49	favorable	DA x3	midostaurin	CR	Negative
	3	71	adverse	DA x2	midostaurin, gilteritinib	CR	Negative
	4	17	intermediate	MEC x1, ADXC x1	sorafenib	PR	Positive
	5	83	adverse	Aza x22	No	sAML	Positive
	6	48	adverse	DA x2	midostaurin	R	Positive
	7	35	intermediate	DA x1	midostaurin		
	8	37	intermediate	DA x1, ACE x2	midostaurin	R	Positive
	9	26	adverse	DA x1, ACE x1	midostaurin	PR	Positive
	10	49	intermediate	Unknown -> HSCT Relapse	midostaurin	R	Positive
	11	68	intermediate	AzaVen x 3 -> HSCT MRD + -> sorafenib -> Relapse	sorafenib	R	Positive
	12	62	intermediate	DA x1	midostaurin	PR	Positive
	13	74	favorable	DA x3	midostaurin	CR	Negative
2	14	81	intermediate	AzaVen x1	No	PR	Positive
	15	75	intermediate	AzaVen x1	No	PR	Positive
	16	77	adverse	DA x3 -> CR -> R	midostaurin	R	Positive
	17	64	intermediate	DA x1	midostaurin	CR	Not measured
	18	39	intermediate	IA x1 , ACE x1	midostaurin, gilteritinib	PR	Not measured
	19	77	intermediate	AzaVen x2	No	PR	Positive
	20	65	adverse	Aza x1, DA x2 -> HSCT -> R -> Aza x2 -> rising FLT3 MRD	midostaurin	R	Positive
	21	47	adverse	DA x1, ACE x1 -> HSCT -> R -> FLAVIda	No	CR	Negative
	22	83	adverse	Aza x7 -> CR -> MDS progression	No	R	Positive
3	23	73	adverse	AzaVen x1	No	sAML	Negative
	24	46	intermediate	DA x3	midostaurin	CR	Negative
	25	77	adverse	AzaVen x1	No	sAML	Not measured
	26	76	intermediate	CLAV x2	gilteritinib	CR	Negative
	27	72	intermediate	DA x2	midostaurin	CR	Negative
	28	76	intermediate	DA x3 -> HSCT -> AzaVen x2	gilteritinib	PR	Positive
	29	38	intermediate	DA x1, FAIda -> HSCT	midostaurin	R	Positive

Supplemental Table S3. Patients' characteristics in the patient group that received GILT triplet therapy as bridging to HSCT vs the remaining patients.

		Bridging to HSCT	Remaining patients
Number of patients		8<br>n (%)	21<br>n (%)
Age, median (range)		38 (17-64)	74 (38-83)
ECOG n (%)	0	3 (37.5)	7 (33.3)
	1	4 (50.0)	9 (42.9)
	2	1 (12.5)	1 (4.8)
	3	0	4 (19.0)
ELN 2022 Cytogenic risk group n (%)	Favorable	0	2 (9.5)
	Intermediate	6 (75.0)	10 (47.6)
	Adverse	2 (25.0)	9 (42.9)
Prior HSCT	n (%)	1 (12.5)	4 (19.0)
Lines of prior therapy n (%)	1	4 (50.0)	16 (76.2)
	2	3 (37.5)	2 (9.5)
	>2	1 (12.5)	3 (14.3)
Disease state at start of triplet therapy	CR/MLFS	1 (12.5)	9 (42.9)
	PR/NR	5 (62.5)	4 (19.0)
	R/sAML	2 (25.0)	8 (38.1)
Prior FLT3 TKI	n (%)	8 (100.0)	12 (57.1)

Supplemental Figure S2. Swimmer plot with MRD measurements

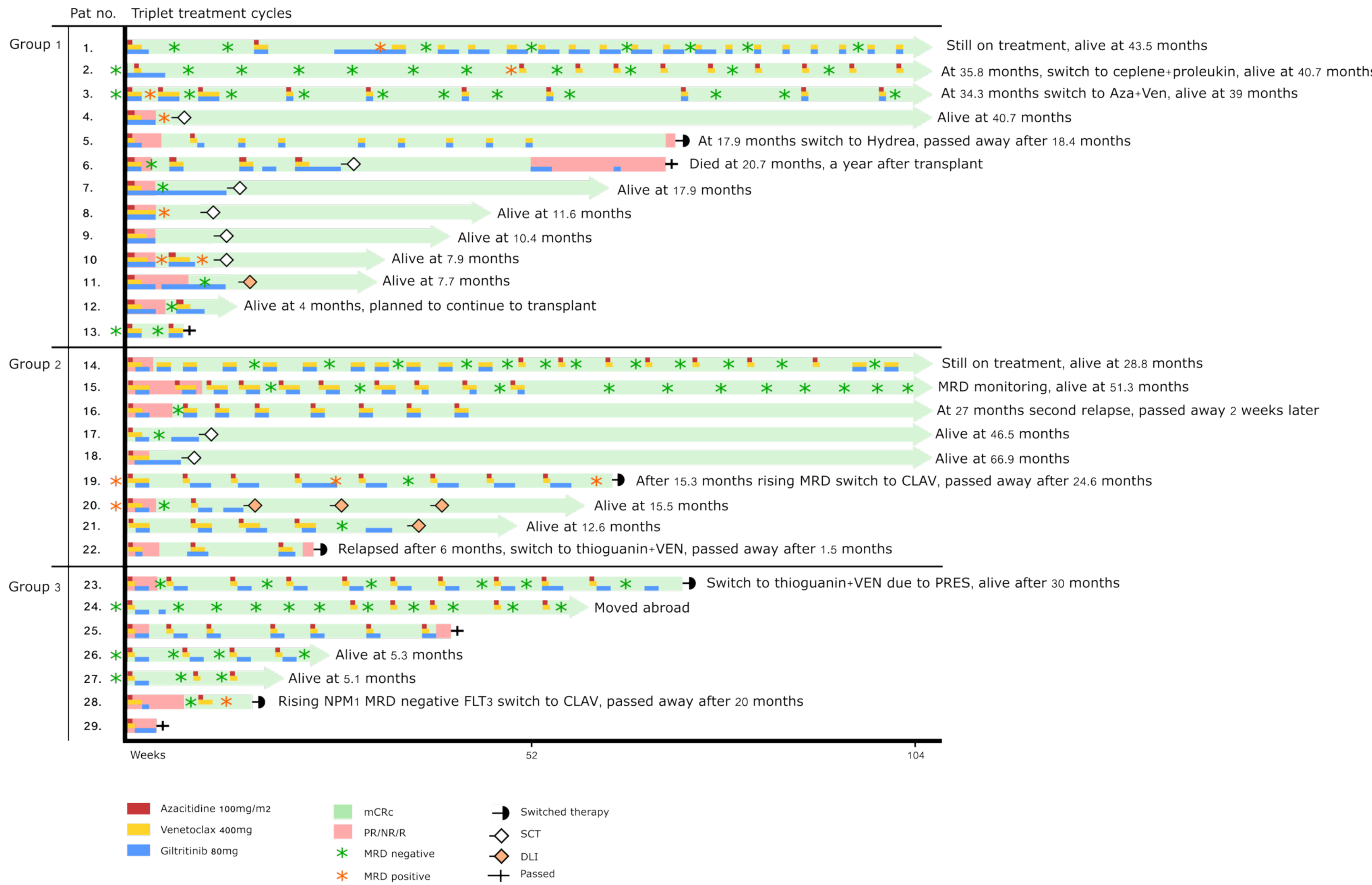

Supplemental Figure S3. OS and EFS stratified by subsequent HSCT for the three Groups. "HSCT" consists of transplanted patients from all 3 groups, where as non-transplanted patients are reported in stratified curves based on treatment schedule.

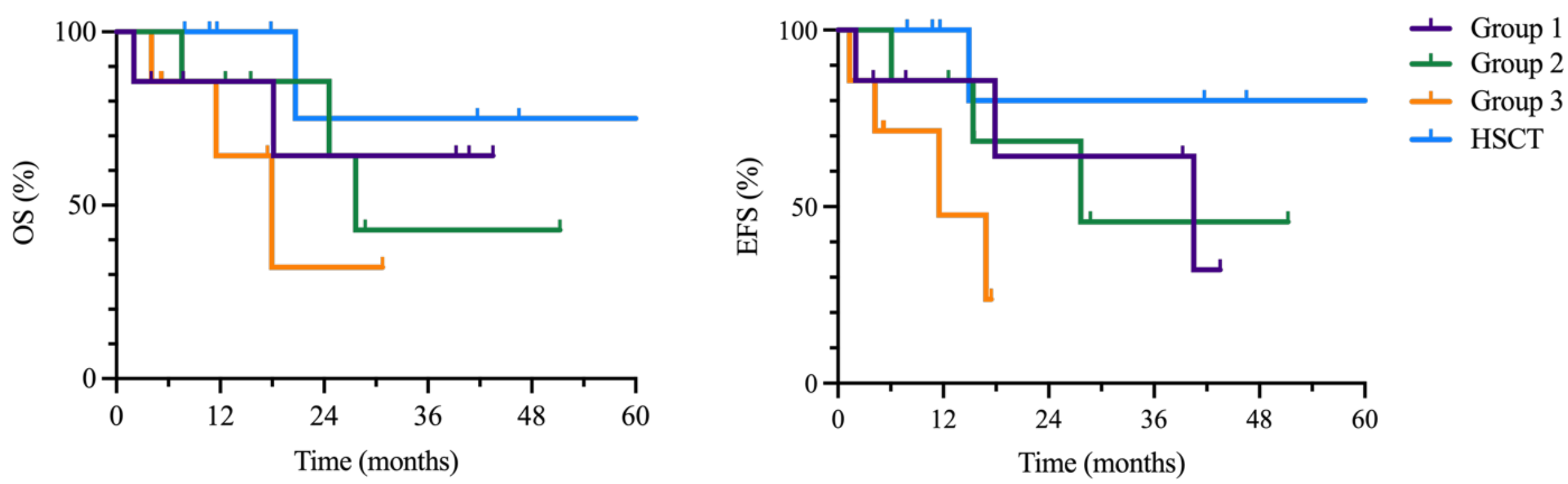

Supplemental Figure S4. Average dose per cycle per Group with AZA, VEN and GILT. Abbreviations: AZA, azacitidine; VEN, venetoclax; GILT, gilteritinib.

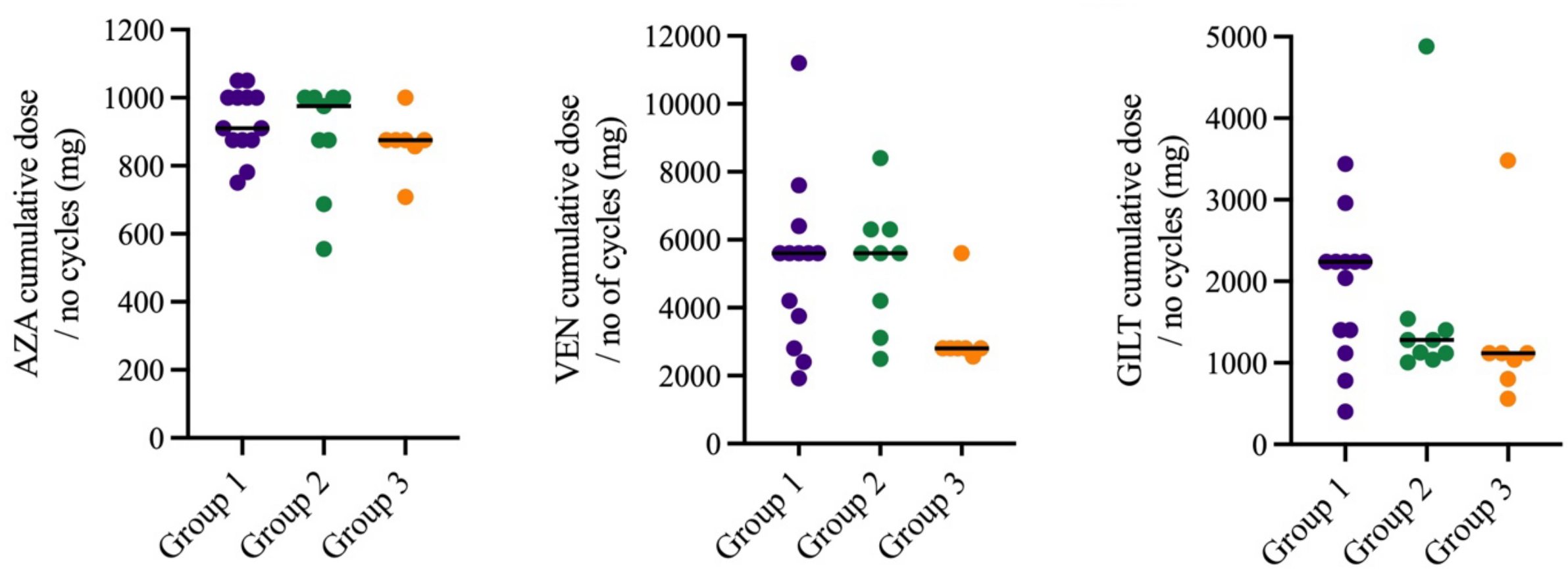

Supplemental Figure S5. Number of GILT triplet therapy per Group.

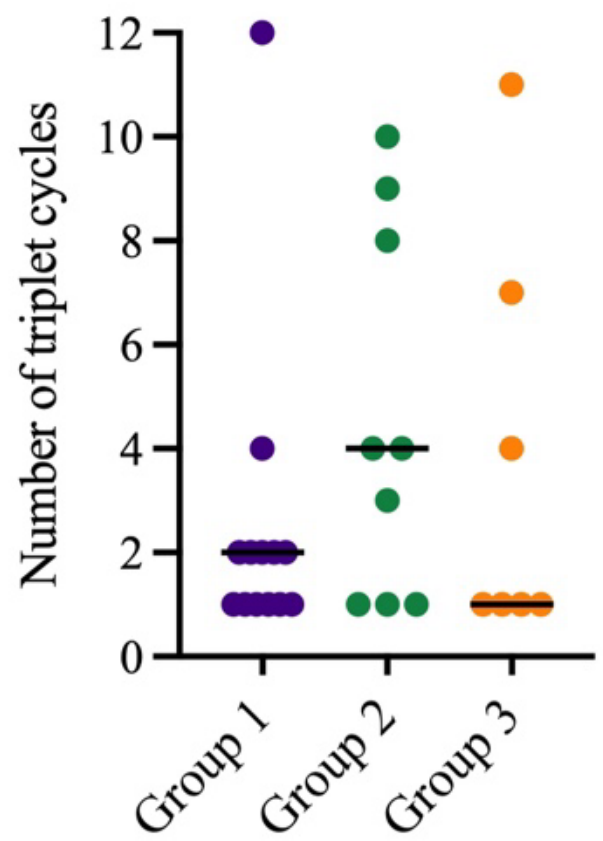

Supplemental Table S4. Adverse Events

AE after cycle	Group 1	Group 2	Group 3
Cycle 1	Febrile neutropenia n=9 (69.2%)<br>Infection n=3 (23.1%)<br>Hepatotoxicity n=1 (7.7%)	Febrile neutropenia n= 1 (11.1%)<br>Infection n=2 (22.2%)	Febrile neutropenia n=0<br>Infection n=1 (14.3%)<br>Tumor lysis n=1 (14.3%)<br>Heart failure n=1 (14.3%)
Cycle 2 until 4	Febrile neutropenia n=2 (33.3%)<br>Febrile sepsis n=1 (16.6%)<br>Infection n=1 (16.6%)<br>GI bleed n=1 (33.3%)<br>Atrial fibrillation n=1 (33.3%)	Infection n=1 (33.3%)	Febrile neutropenia n=1 (33.3%)<br>PRES n=1

Supplemental Figure S6. Relapsed and refractory patients, combining the data from the R/sAML and the PR/NR cohort with a total of 19 patients.

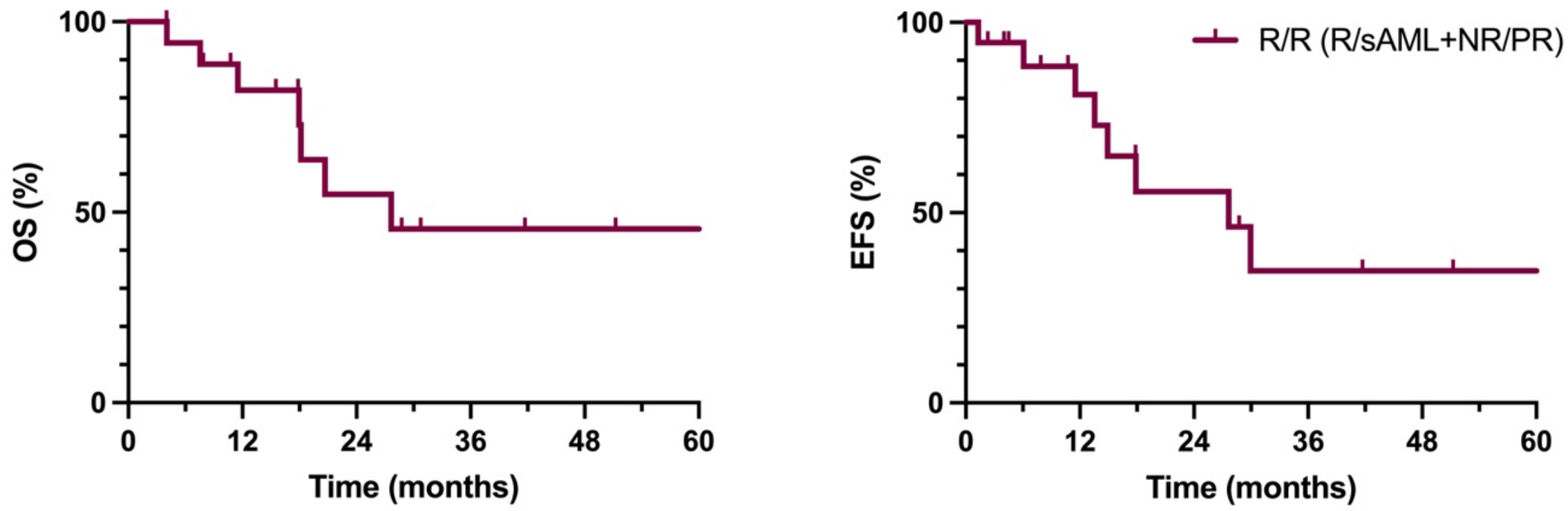

n=19 with median OS and median EFS 27.6 months.

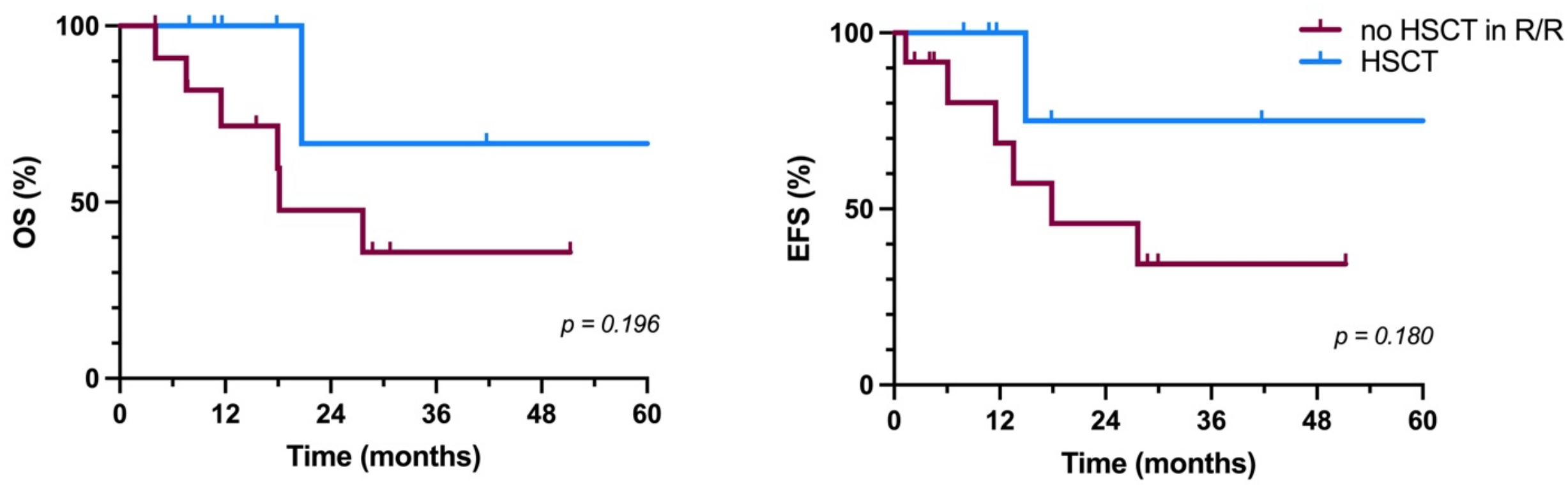

n=19 with median OS 18.1 months and median EFS 17.9 months in non-transplanted patients.
